# Supplementary material for: Anillin/Mid1p interacts with the ESCRT-associated protein Vps4p and mitotic kinases to regulate cytokinesis in fission yeast
Source: Cell Cycle. 2021 Aug 12;20(18):1845–60. doi: 10.1080/15384101.2021.1962637 (PMC8525990; doi:10.1080/15384101.2021.1962637)
Supplement: Supplemental Material [file KCCY_A_1962637_SM1576.zip › Supplementary information/Rezig et al S1 Table.docx]

S1 Table. *S. pombe* strains used in this study. "GG" number refers to the laboratory reference collection. All strains *ade*^-^, unless indicated.

| **GG No.** | **Genotype Annotation** |
| --- | --- |
| 1 | h^-^ 972 wild-type (W-T) |
| 397 | h*^+^* *ade6-210 leu1-32 ura4-D18* |
| 400 | h^-^ *ade6-216 leu1-32 ura4-D18* |
| 1129 | h^-^ *mid1*::*ura4^+^* *ura4-D18 leu1-32* *mid1*∆ |
| 1167 | h^-^ *plo1-ts35 ura4-D18 leu1-32 plo-ts35* |
| 1347 | pmid-*mid1*-4GFP (integrated; pAP221; *leu1^+^*) Mid1p-GFP  h^-^ *dmf1*::*ura4^+^* *ura4*-D18 *leu1-32* |
| 1349 | pmid-NLS*-*mid1*-GFP (integrated; pAP167#2; *leu1^+^*)  h^-^ *dmf1*::*ura4^+^* *ura4-D18 leu1-32* |
| 1384 | pAM19 (D450-506 *mid1*:*12myc*:*leu1^+^*)  h^-^ *dmf1*::*ura4^+^ura4-D18 leu1-32* |
| 1388 | pAM23 (*NLS*mid1*:*12myc*:*leu1^+^*)  h^-^ *dmf1*::*ura4^+^* *ura4-D18 leu1-32* |
| 1554 | h*^+^* *dmf1*::*kanMX4* *ura4-D18 leu1-32 ade^+^* *mid1*∆ |
| 1622 | h^-^ *vps4*::*ura4^+^* *leu1-32 ura4-D18* *ade^+^* *vps4*∆ |
| 2417 | h*^+^* *ark1-T11*<<kanR *leu1-32 ade^+^*  *ark1-T11* |
| 2432 | h*^+^* *ark1-T8*<<kanR *leu1-32 ade^+^*  *ark1-T8* |
| 2451 | h+ *ark1-T11*<<kanR *ura4-C190T leu1-32* *ark1-T11* |
| 2673 | h*^+^* *vps4*::*ura4^+^* *leu1-32 ura4-D18 ade^+^*  *vps4*∆ |
| 2674 | pmid-*mid1*-4GFP (integrated; pAP221; *leu1^+^*) *vps4*∆ Mid1p-GFP  h^-^ *dmf1*::*ura4^+^ura4-D18? leu1-32 vps4*::*ura4^+^* |
| 2709 | pmid-*mid1*-4GFP (integrated; pAP221; *leu1^+^*) *vps4*∆ Mid1p-GFP  h^-^ *dmf1*::*ura4^+^ura4-D18? leu1-32 vps4*::*ura4^+^* |
| 2886 | pmid-*mid1*-4GFP (integrated; pAP221; *leu1^+^*) *ark1-T11* Mid1p-GFP  h^-^ *dmf1*::*ura4^+^ura4-D18? leu1-32 ark1*-*T11*<<kanR |
| 2922 | pmid-mid1-4GFP (integrated; pAP221; *leu1^+^*) *ark1-T8* Mid1p-GFP  h*^+^* *dmf1*::*ura4^+^* *ura4-D18 leu1-32 ark1*-*T8*<<kanR |
| 3100 | NLS* *mid1*:*12myc*:*leu1^+^*  h^-^ *dmf1*::*ura4^+^ vps4*::*ura4^+^* *ura4-D18 leu1-32* |
| 3107 | D450-506 *mid1*:*12myc*:*leu1^+^*  h^-^ *dmf1*::*ura4^+^ vps4*::*ura4^+^* *ura4-D18 leu1-32* |
| 3181 | pJK148:*mid1^+^* (wild-type) *mid1*∆ pJK148:*mid1^+^* h^-^ *mid1*::*ura4^+^* *ura4-D18 leu1-32* |
| 3185 | pJK148:*mid1* S523 to A523 h^-^ *mid1*::*ura4^+^* *ura4-D18 leu1-32* |
| 3189 | pJK148:*mid1* S523 to D523 h^-^ *mid1*::*ura4^+^* *ura4-D18 leu1-32* |
| 3193 | pJK148:*mid1* S531 to A531 h^-^ *mid1*::*ura4^+^* *ura4-D18 leu1-32* |
| 3197 | pJK148:*mid1* S531 to D531 h^-^ *mid1*::*ura4^+^* *ura4-D18 leu1-32* |
| 3201 | pJK148:*mid1* S523+S531 to A523+A531  h^-^ *mid1*::*ura4^+^* *ura4-D18 leu1-32* |
| 3205 | pJK148:*mid1* S523+S531 to D523+D531  h^-^ *mid1*::*ura4^+^* *ura4-D18 leu1-32* |
| 3218 | pJK148:*mid1* S523 to A523  h^?^ *mid1*::*ura4^+^* *ark1*-*T11*<<kanR *ade^+^* |
| 3224 | pJK148:*mid1* S523 to A523  h^?^ *mid1*::*ura4^+^ vps4*::*ura4+ura4-D18 leu1-32* |
| 3230 | pJK148:*mid1* S523 to D523  h^?^ *mid1*::*ura4^+^ ark1*-*T11*<<kanR *ura4-C190T leu1-32* |
| 3235 | pJK148:*mid1* S531 to A531  h^?^ *mid1*::*ura4^+^ ark1*-*T11*<<kanR *ura4-C190T leu1-32* |
| 3239 | pJK148:*mid1* S531 to D531  h^?^ *mid1*::*ura4^+^ ark1-T11*<<kanR *ura4-C190T leu1-32* |
| 3242 | pJK148:*mid1* S523 to A523+S531 to A531  h^?^ *mid1*::*ura4^+^ ark1-T11<*<kanR *ura4-C190T leu1-32* |
| 3246 | pJK148:*mid1* S523+S531 to D523+D531  h^?^ *mid1*::*ura4^+^ ark1-T11*<<kanR *ura4-C190T leu1-32* |
| 3249 | pJK148:*mid1* S523 to A523  h^?^ *mid1*::*ura4^+^ vps4*::*ura4+ura4-D18 leu1-32* |
| 3250 | pJK148:*mid1* (wild-type) *mid1*∆ pJK148:*mid1^+^ ark1-T11*  h^?^ *mid1*::*ura4^+^ ark1-T11*<<kanR *ura4-C190T leu1-32* |
| 3255 | pJK148:*mid1* S523 to A523  h^?^ *mid1*::*ura4^+^ vps4*::*ura4+ura4-D18 leu1-32* |
| 3256 | pJK148:*mid1* S523 to A523  h^?^ *mid1*::*ura4^+^ vps4*::*ura4+ura4-D18 leu1-32* |
| 3257 | pJK148:*mid1* S523+S531 to D523+D531  h^-^ *mid1*::*ura4^+^ vps4*::*ura4+ ura4-D18 leu1-32* |
| 3258 | pJK148:*mid1* S523+S531 to D523+D531  h^-^ *mid1*::*ura4^+^ vps4*::*ura4+ ura4-D18 leu1-32* |
| 3260 | pJK148:*mid1* S523+S531 to A523+A531  h^?^ *mid1*::*ura4^+^ vps4*::*ura4^+^ ura4-D18 leu1-32* |
| 3264 | pJK148:mid1 S531 to D531  h^?^ *mid1*::*ura4^+^ vps4*::*ura4^+^ ura4-D18 leu1-32* |
| 3267 | pJK148:*mid1* S167 to A167 h^-^ *mid1*::*ura4^+^* *ura4-D18 leu1-32* |
| 3271 | pJK148:*mid1* S167 to D167 h^-^ *mid1*::*ura4^+^* *ura4-D18 leu1-32* |
| 3275 | pJK148:*mid1* S328 to A328 h^-^ *mid1*::*ura4^+^* *ura4-D18 leu1-32* |
| 3280 | pJK148:*mid1* S328 to D328  h^-^ *mid1*::*ura4^+^* *ura4-D18 leu1-32* |
| 3283 | pJK148:*mid1* S331 to A331 h^-^ *mid1*::*ura4^+^* *ura4-D18 leu1-32* |
| 3290 | pJK148:*mid1* S331 to D331 h^-^ *mid1*::*ura4^+^ ura4-D18 leu1-32* |
| 3291 | pJK148:*mid1* S332 to A332 h^-^ *mid1*::*ura4^+^* *ura4-D18 leu1-32* |
| 3295 | pJK148:*mid1* S332 to D332 h^-^ *mid1*::*ura4^+^* *ura4-D18 leu1-32* |
| 3299 | pJK148:*mid1* S167+S328+S331+S332 to A167+A328+A331+A332  h^-^ *mid1*::*ura4^+^* *ura4-D18 leu1-32* |
| 3305 | pJK148:*mid1* S167+S328+S331+S332 to D167+D328+D331+D332  h^-^ *mid1*::*ura4^+^* *ura4-D18 leu1-32* |
| 3307 | pJK148:*mid1* S167+S328+S331+S332+S523+S531 to A167+A328+A331+A332+A523+A531  h^-^ *mid1*::*ura4^+^* *ura4-D18 leu1-32* |
| 3311 | pJK148:*mid1* S167+S328+S331+S332+S523+S531 to D167+D328+D331+D332+D523+D531  h^-^ *mid1*::*ura4^+^* *ura4-D18 leu1-32* |
| 3315 | pJK148:*mid1* S523 to D523  h^?^ *mid1*::*ura4^+^ vps4*::*ura4+ ura4-D18 leu1-32* |
| 3316 | pJK148:*mid1* S523 to D523  h^?^ *mid1*::*ura4^+^ vps4*::*ura4+ ura4-D18 leu1-32* |
| 3317 | pJK148:*mid1* S523 to D523  h^?^ *mid1*::*ura4^+^ vps4*::*ura4+ ura4-D18 leu1-32* |
| 3318 | pJK148:*mid1* S531 to A531  h^?^ *mid1*::*ura4^+^ vps4*::*ura4+ ura4-D18 leu1-32* |
| 3319 | pJK148:*mid1* S531 to A531  h^?^ *mid1*::*ura4^+^ vps4*::*ura4+ ura4-D18 leu1-32* |
| 3320 | pJK148:*mid1* S531 to A531  h^?^ *mid1*::*ura4^+^ vps4*::*ura4+ ura4-D18 leu1-32* |
| 3321 | pJK148:*mid1* S167 to A167  h^?^ *mid1*::*ura4^+^ ark1*-*T11*<<kanR ura4-C190T *leu1-32* |
| 3322 | pJK148:*mid1* S167 to A167  h^?^ *mid1*::*ura4^+^ ark1*-*T11*<<kanR ura4-C190T *leu1-32* |
| 3323 | pJK148:*mid1* S167 to A167  h^?^ *mid1*::*ura4^+^ ark1*-*T11*<<kanR ura4-C190T *leu1-32* |
| 3324 | JK148:*mid1* S167 to D167  h^?^ *mid1*::*ura4^+^ ark1*-*T11*<<kanR *ura4-C190T leu1-32* |
| 3325 | JK148:*mid1* S167 to D167  h^?^ *mid1*::*ura4^+^ ark1*-*T11*<<kanR *ura4-C190T leu1-32* |
| 3326 | JK148:*mid1* S167 to D167  h^?^ *mid1*::*ura4^+^ ark1*-*T11*<<kanR *ura4-C190T leu1-32* |
| 3327 | pJK148:*mid1* S328 to A328 h^?^ *mid1*::*ura4^+^ ark1*-*T11*<<kanR *ura4-C190T leu1-32* |
| 3328 | pJK148:*mid1* S328 to A328 h^?^ *mid1*::*ura4^+^ ark1*-*T11*<<kanR *ura4-C190T leu1-32* |
| 3329 | pJK148:*mid1* S328 to A328 h^?^ *mid1*::*ura4^+^ ark1*-*T11*<<kanR *ura4-C190T leu1-32* |
| 3330 | pJK148:*mid1* S328 to D328 h^?^ *mid1*::*ura4^+^ ark1*-*T11*<<kanR *ura4*-C190T *leu1-32* |
| 3331 | pJK148:*mid1* S328 to D328 h^?^ *mid1*::*ura4^+^ ark1*-*T11*<<kanR *ura4*-C190T *leu1-32* |
| 3332 | pJK148:*mid1* S328 to D328 h^?^ *mid1*::*ura4^+^ ark1*-*T11*<<kanR *ura4*-C190T *leu1-32* |
| 3333 | pJK148:*mid1* S331 to A331 h^?^ *mid1*::*ura4^+^ ark1*-*T11*<<kanR *ura4-C190T leu1-32* |
| 3336 | pJK148:*mid1* S331 to D331 h^?^ *mid1*::*ura4^+^ ark1*-*T11*<<kanR *ura4-C190T leu1-32* |
| 3339 | pJK148:*mid1* S332 to A332 h^?^ *mid1*::*ura4^+^ ark1*-*T11*<<kanR *ura4-C190T leu1-32* |
| 3340 | pJK148:*mid1* S332 to A332 h^?^ *mid1*::*ura4^+^ ark1*-*T11*<<kanR *ura4-C190T leu1-32* |
| 3341 | pJK148:*mid1* S332 to A332 h^?^ *mid1*::*ura4^+^ ark1*-*T11*<<kanR *ura4-C190T leu1-32* |
| 3342 | pJK148:*mid1* S332 to D332 h^?^ *mid1*::*ura4^+^ ark1*-*T11*<<kanR *ura4-C190T leu1-32* |
| 3343 | pJK148:*mid1* S332 to D332 h^?^ *mid1*::*ura4^+^ ark1*-*T11*<<kanR *ura4-C190T leu1-32* |
| 3344 | pJK148:*mid1* S332 to D332 h^?^ *mid1*::*ura4^+^ ark1*-*T11*<<kanR *ura4-C190T leu1-32* |
| 3345 | pJK148:*mid1* S167+S328+S331+S332 to A167+A328+A331+A332  h^?^ *mid1*::*ura4^+^ ark1*-*T11*<<kanR *ura4-C190T leu1-32* |
| 3346 | pJK148:*mid1* S167+S328+S331+S332 to A167+A328+A331+A332  h^?^ *mid1*::*ura4^+^ ark1*-*T11*<<kanR *ura4-C190T leu1-32* |
| 3347 | pJK148:*mid1* S167+S328+S331+S332 to A167+A328+A331+A332  h^?^ *mid1*::*ura4^+^ ark1*-*T11*<<kanR *ura4-C190T leu1-32* |
| 3349 | pJK148:*mid1* S167+S328+S331+S332 to D167+D328+D331+D332  h^?^ *mid1*::*ura4^+^ ark1*-*T11*<<kanR *ura4-C190T leu1-32* |
| 3350 | pJK148:*mid1* S167+S328+S331+S332 to D167+D328+D331+D332  h^?^ *mid1*::*ura4^+^ ark1*-*T11*<<kanR *ura4-C190T leu1-32* |
| 3351 | pJK148:*mid1* S167+S328+S331+S332 to D167+D328+D331+D332  h^?^ *mid1*::*ura4^+^ ark1*-*T11*<<kanR *ura4-C190T leu1-32* |
| 3352 | pJK148:*mid1* S167+S328+S331+S332+S523+S531 to A167+A328+A331+A332+A523+A531  h^?^ *mid1*::*ura4^+^ ark1-T11*<<kanR *ura4-C190T leu1-32* |
| 3353 | pJK148:*mid1* S167+S328+S331+S332+S523+S531 to A167+A328+A331+A332+A523+A531  h^?^ *mid1*::*ura4^+^ ark1-T11*<<kanR *ura4-C190T leu1-32* |
| 3354 | pJK148:*mid1* S167+S328+S331+S332+S523+S531 to A167+A328+A331+A332+A523+A531  h^?^ *mid1*::*ura4^+^ ark1-T11*<<kanR *ura4-C190T leu1-32* |
| 3355 | pJK148:*mid1* S167+S328+S331+S332+S523+S531 to D167+D328+D331+D332+D523+D531  h^?^ *mid1*::*ura4^+^ ark1*-*T11*<<kanR *ura4-C190T leu1-32* |
| 3356 | pJK148:*mid1* S167+S328+S331+S332+S523+S531 to D167+D328+D331+D332+D523+D531  h^?^ *mid1*::*ura4^+^ ark1*-*T11*<<kanR *ura4-C190T leu1-32* |
| 3357 | pJK148:*mid1* S167+S328+S331+S332+S523+S531 to D167+D328+D331+D332+D523+D531  h^?^ *mid1*::*ura4^+^ ark1*-*T11*<<kanR *ura4-C190T leu1-32* |
| 3375 | pJK148:*mid1* S167 to A167  h^?^ *mid1*::*ura4^+^ plo1-ts35 ura4-D18 leu1-32* |
| 3376 | pJK148:*mid1* S167 to A167  h^?^ *mid1*::*ura4^+^ plo1-ts35 ura4-D18 leu1-32* |
| 3378 | pJK148:mid1 S167 to D167  h^?^ *mid1*::*ura4^+^ plo1-ts35 ura4-D18 leu1-32* |
| 3377 | pJK148:mid1 S167 to D167  h^?^ *mid1*::*ura4^+^ plo1-ts35 ura4-D18 leu1-32* |
| 3378 | pJK148:mid1 S167 to D167  h^?^ *mid1*::*ura4^+^ plo1-ts35 ura4-D18 leu1-32* |
| 3379 | pJK148:*mid1* S167 to A167  h^?^ *mid1*::*ura4^+^ plo1-ts35 ura4-D18 leu1-32* |
| 3381 | pJK148:*mid1* S167 to A167  h^?^ *mid1*::*ura4^+^ plo1-ts35 ura4-D18 leu1-32* |
| 3383 | pJK148:*mid1* S167 to A167  h^?^ *mid1*::*ura4^+^ vps4*::*ura4^+^ ura4-D18 leu1-32* |
| 3384 | pJK148:*mid1* S167 to A167  h^?^ *mid1*::*ura4^+^ vps4*::*ura4^+^ ura4-D18 leu1-32* |
| 3452 | pJK148:*mid1* S167 to A167  h^?^ *mid1*::*ura4^+^ vps4*::*ura4^+^ ura4-D18 leu1-32* |
| 3386 | pJK148:*mid1* S328 to D328  h^?^ *mid1*::*ura4^+^ plo1-ts35 ura4-D18 leu1-32* |
| 3387 | pJK148:*mid1* S328 to D328  h^?^ *mid1*::*ura4^+^ plo1-ts35 ura4-D18 leu1-32* |
| 3388 | pJK148:*mid1* S328 to D328  h^?^ *mid1*::*ura4^+^ plo1-ts35 ura4-D18 leu1-32* |
| 3392 | pJK148:*mid1* S328 to A328  h^?^ *mid1*::*ura4^+^ plo1-ts35 ura4-D18 leu1-32* |
| 3393 | pJK148:*mid1* S328 to A328  h^?^ *mid1*::*ura4^+^ plo1-ts35 ura4-D18 leu1-32* |
| 3394 | pJK148:*mid1* S328 to A328  h^?^ *mid1*::*ura4^+^ plo1-ts35 ura4-D18 leu1-32* |
| 3397 | pJK148:*mid1* S331 to A331  h^?^ *mid1*::*ura4^+^ plo1-ts35 ura4-D18 leu1-32* |
| 3398 | pJK148:*mid1* S331 to A331  h^?^ *mid1*::*ura4^+^ plo1-ts35 ura4-D18 leu1-32* |
| 3399 | pJK148:*mid1* S331 to A331  h^?^ *mid1*::*ura4^+^ plo1-ts35 ura4-D18 leu1-32* |
| 3400 | pJK148:*mid1* S332 to A332  h^?^ *mid1*::*ura4^+^ plo1-ts35 ura4-D18 leu1-32* |
| 3401 | pJK148:*mid1* S332 to A332  h^?^ *mid1*::*ura4^+^ plo1-ts35 ura4-D18 leu1-32* |
| 3402 | pJK148:*mid1* S332 to A332  h^?^ *mid1*::*ura4^+^ plo1-ts35 ura4-D18 leu1-32* |
| 3403 | pJK148:*mid1* S523 to D523  h^-^ *mid1*::*ura4^+^ plo1-ts35 ura4-D18 leu1-32* |
| 3404 | pJK148:*mid1* S523 to D523  h^-^ *mid1*::*ura4^+^ plo1-ts35 ura4-D18 leu1-32* |
| 3405 | pJK148:*mid1* S523 to D523  h^-^ *mid1*::*ura4^+^ plo1-ts35 ura4-D18 leu1-32* |
| 3407 | pJK148:*mid1* S523 to A523  h^-^ *mid1*::*ura4^+^ plo1-ts35 ura4-D18 leu1-32* |
| 3408 | pJK148:*mid1* S523 to A523  h^-^ *mid1*::*ura4^+^ plo1-ts35 ura4-D18 leu1-32* |
| 3409 | pJK148:*mid1* S523 to A523  h^-^ *mid1*::*ura4^+^ plo1-ts35 ura4-D18 leu1-32* |
| 3411 | pJK148:*mid1* S332 to D332  h^-^ *mid1*::*ura4^+^ plo1-ts35 ura4-D18 leu1-32* |
| 3412 | pJK148:*mid1* S332 to D332  h^-^ *mid1*::*ura4^+^ plo1-ts35 ura4-D18 leu1-32* |
| 3413 | pJK148:*mid1* S332 to D332  h^-^ *mid1*::*ura4^+^ plo1-ts35 ura4-D18 leu1-32* |
| 3415 | pJK148:*mid1* S531 to A531  h^-^ *mid1*::*ura4^+^ plo1-ts35 ura4-D18 leu1-32* |
| 3416 | pJK148:*mid1* S531 to A531  h^-^ *mid1*::*ura4^+^ plo1-ts35 ura4-D18 leu1-32* |
| 3417 | pJK148:*mid1* S531 to A531  h^-^ *mid1*::*ura4^+^ plo1-ts35 ura4-D18 leu1-32* |
| 3419 | pJK148:*mid1* S167+S328+S331+S332 to A167+A328+A331+A332  h^-^ *mid1*::*ura4^+^ plo1-ts35 ura4-D18 leu1-32* |
| 3420 | pJK148:*mid1* S167+S328+S331+S332 to A167+A328+A331+A332  h^-^ *mid1*::*ura4^+^ plo1-ts35 ura4-D18 leu1-32* |
| 3421 | pJK148:*mid1* S167+S328+S331+S332 to A167+A328+A331+A332  h^-^ *mid1*::*ura4^+^ plo1-ts35 ura4-D18 leu1-32* |
| 3422 | pJK148:*mid1* S167+S328+S331+S332 to D167+D328+D331+D332  h^-^ *mid1*::*ura4^+^ plo1-ts35 ura4-D18 leu1-32* |
| 3423 | pJK148:*mid1* S167+S328+S331+S332 to D167+D328+D331+D332  h^-^ *mid1*::*ura4^+^ plo1-ts35 ura4-D18 leu1-32* |
| 3424 | pJK148:*mid1* S167+S328+S331+S332 to D167+D328+D331+D332  h^-^ *mid1*::*ura4^+^ plo1-ts35 ura4-D18 leu1-32* |
| 3425 | pJK148:*mid1* S167+S328+S331+S332+S523+S531 to  A167+A328+A331+A332+A523+A531  h^-^ *mid1*::*ura4^+^ plo1-ts35 ura4-D18 leu1-32* |
| 3426 | pJK148:*mid1* S167+S328+S331+S332+S523+S531 to  A167+A328+A331+A332+A523+A531  h^-^ *mid1*::*ura4^+^ plo1-ts35 ura4-D18 leu1-32* |
| 3427 | pJK148:*mid1* S167+S328+S331+S332+S523+S531 to  A167+A328+A331+A332+A523+A531  h^-^ *mid1*::*ura4^+^ plo1-ts35 ura4-D18 leu1-32* |
| 3428 | pJK148:*mid1* S531 to D531  h^-^ *mid1*::*ura4^+^ plo1-ts35 ura4-D18 leu1-32* |
| 3429 | pJK148:*mid1* S531 to D531  h^-^ *mid1*::*ura4^+^ plo1-ts35 ura4-D18 leu1-32* |
| 3430 | pJK148:*mid1* S531 to D531  h^-^ *mid1*::*ura4^+^ plo1-ts35 ura4-D18 leu1-32* |
| 3437 | pJK148:*mid1*^+^ (wild-type) *mid1*∆ pJK148:*mid1^+^ plo1-ts35*  h^-^ *mid1::ura4*^+^ *plo1-ts35 ura4-D18 leu1-32* |
| 3438 | pJK148:*mid1*^+^ (wild-type) *mid1*∆ pJK148:*mid1^+^ plo1-ts35*  h^-^ *mid1::ura4*^+^ *plo1-ts35 ura4-D18 leu1-32* |
| 3439 | pJK148:*mid1*^+^ (wild-type) *mid1*∆ pJK148:*mid1^+^ plo1-ts35*  h^-^ *mid1::ura4*^+^ *plo1-ts35 ura4-D18 leu1-32* |
| 3441 | pJK148:*mid1* S167+S328+S331+S332+S523+S531 to  D167+D328+D331+D332+D523+D531  h- *mid1::ura4*^+^ *plo1-ts35 ura4-D18 leu1-32* |
| 3442 | pJK148:*mid1* S167+S328+S331+S332+S523+S531 to  D167+D328+D331+D332+D523+D531  h- *mid1::ura4*^+^ *plo1-ts35 ura4-D18 leu1-32* |
| 3443 | pJK148:*mid1* S167+S328+S331+S332+S523+S531 to  D167+D328+D331+D332+D523+D531  h- *mid1::ura4*^+^ *plo1-ts35 ura4-D18 leu1-32* |
| 3444 | pJK148:*mid1* S523 to A523 + S531 to A531  h^-^ *mid1::ura4*^+^ *plo1-ts35 ura4-D18 leu1-32* |
| 3445 | pJK148:*mid1* S523 to A523 + S531 to A531  h^-^ *mid1::ura4*^+^ *plo1-ts35 ura4-D18 leu1-32* |
| 3446 | pJK148:*mid1* S523 to A523 + S531 to A531  h^-^ *mid1::ura4*^+^ *plo1-ts35 ura4-D18 leu1-32* |
| 3447 | pJK148:*mid1* S523 to D523 + S531 to D531  h- *mid1::ura4+ plo1-ts35 ura4-D18 leu1-32* |
| 3448 | pJK148:*mid1* S523 to D523 + S531 to D531  h- *mid1::ura4+ plo1-ts35 ura4-D18 leu1-32* |
| 3449 | pJK148:*mid1* S523 to D523 + S531 to D531  h- *mid1::ura4+ plo1-ts35 ura4-D18 leu1-32* |
| 3450 | pJK148:*mid1* S167 to A167  h^?^ *mid1*::*ura4^+^ vps4*::*ura4+ ura4-D18 leu1-32* |
| 3451 | pJK148:*mid1* S167 to A167  h^?^ *mid1*::*ura4^+^ vps4*::*ura4+ ura4-D18 leu1-32* |
| 3452 | pJK148:*mid1* S167 to A167  h^?^ *mid1*::*ura4^+^ vps4*::*ura4+ ura4-D18 leu1-32* |
| 3456 | pJK148:*mid1* S328 to A328  h^?^ *mid1*::*ura4^+^ vps4*::*ura4+ ura4-D18 leu1-32* |
| 3457 | pJK148:*mid1* S328 to A328  h^?^ *mid1*::*ura4^+^ vps4*::*ura4+ ura4-D18 leu1-32* |
| 3458 | pJK148:*mid1* S328 to A328  h^?^ *mid1*::*ura4^+^ vps4*::*ura4+ ura4-D18 leu1-32* |
| 3461 | pJK148:*mid1* S167+S328+S331+S332 to D167+D328+D331+D332  h^?^ *mid1*::*ura4^+^ vps4*::*ura4+ ura4-D18 leu1-32* |
| 3463 | pJK148:*mid1* S167+S328+S331+S332+S523+S531 to  D167+D328+D331+D332+D523+D531  h^?^ *mid1*::*ura4^+^ vps4*::*ura4+ ura4-D18 leu1-32* |
| 3464 | pJK148:*mid1* S167+S328+S331+S332+S523+S531 to  D167+D328+D331+D332+D523+D531  h^?^ *mid1*::*ura4^+^ vps4*::*ura4+ ura4-D18 leu1-32* |
| 3465 | pJK148:*mid1* S167+S328+S331+S332+S523+S531 to  D167+D328+D331+D332+D523+D531  h^?^ *mid1*::*ura4^+^ vps4*::*ura4+ ura4-D18 leu1-32* |
| 3467 | pJK148:*mid1* S167+S328+S331+S332+S523+S531 to  A167+A328+A331+A332+A523+A531  h^?^ *mid1*::*ura4^+^ vps4*::*ura4+ ura4-D18 leu1-32* |
| 3468 | pJK148:*mid1* S167+S328+S331+S332+S523+S531 to  A167+A328+A331+A332+A523+A531 h^?^ *mid1*::*ura4^+^ vps4*::*ura4+ ura4-D18 leu1-32* |
| 3469 | pJK148:*mid1* S332 to D332  h^?^ *mid1*::*ura4^+^ vps4*::*ura4+ ura4-D18 leu1-32* |
| 3470 | pJK148:*mid1* S167+S328+S331+S332+S523+S531 to  A167+A328+A331+A332+A523+A531  h^?^ *mid1*::*ura4^+^ vps4*::*ura4+ ura4-D18 leu1-32* |
| 3473 | pJK148:*mid1* S332 to A332  h^?^ *mid1*::*ura4^+^ vps4*::*ura4+ ura4-D18 leu1-32* |
| 3474 | pJK148:*mid1* S332 to A332  h^?^ *mid1*::*ura4^+^ vps4*::*ura4+ ura4-D18 leu1-32* |
| 3475 | pJK148:*mid1* S332 to A332  h^?^ *mid1*::*ura4^+^ vps4*::*ura4+ ura4-D18 leu1-32* |
| 3478 | pJK148:*mid1* S332 to D332  h^?^ *mid1*::*ura4^+^ vps4*::*ura4+ ura4-D18 leu1-32* |
| 3479 | pJK148:*mid1* S332 to D332  h^?^ *mid1*::*ura4^+^ vps4*::*ura4+ ura4-D18 leu1-32* |
| 3481 | pJK148:*mid1* S328 to D328 h^?^ *mid1*::*ura4^+^ vps4*::*ura4+ ura4-D18 leu1-32* |
| 3482 | pJK148:*mid1* S328 to D328 h^?^ *mid1*::*ura4^+^ vps4*::*ura4+ ura4-D18 leu1-32* |
| 3483 | pJK148:*mid1* S328 to D328 h^?^ *mid1*::*ura4^+^ vps4*::*ura4+ ura4-D18 leu1-32* |
| 3485 | pJK148:*mid1* S331 to A331 h^?^ *mid1*::*ura4^+^ vps4*::*ura4+ ura4-D18 leu1-32* |
| 3486 | pJK148:*mid1*^+^ (wild-type) *mid1*∆ pJK148:*mid1^+^ vps4*∆ h^?^ *mid1*::*ura4^+^ vps4*::*ura4+ ura4-D18 leu1-32* |
| 3488 | pJK148:*mid1*^+^ S167+S328+S331+S332 to A167+A328+A331+A332  h^?^ *mid1*::*ura4^+^ vps4*::*ura4+ ura4-D18 leu1-32* |
| 3489 | pJK148:*mid1* S331 to A331 h^?^ *mid1*::*ura4^+^ vps4*::*ura4+ ura4-D18 leu1-32* |
| 3493 | pJK148:*mid1*^+^ S167+S328+S331+S332 to A167+A328+A331+A332  h^?^ *mid1*::*ura4^+^ vps4*::*ura4+ ura4-D18 leu1-32* |
| 3494 | pJK148:*mid1*^+^ S167+S328+S331+S332 to A167+A328+A331+A332  h^?^ *mid1*::*ura4^+^ vps4*::*ura4+ ura4-D18 leu1-32* |
| 3495 | pJK148:*mid1* S331 to A331 h^?^ *mid1*::*ura4^+^ vps4*::*ura4+ ura4-D18 leu1-32* |
