# Supplementary material for: Anillin/Mid1p interacts with the ESCRT-associated protein Vps4p and mitotic kinases to regulate cytokinesis in fission yeast
Source: Cell Cycle. 2021 Aug 12;20(18):1845–60. doi: 10.1080/15384101.2021.1962637 (PMC8525990; doi:10.1080/15384101.2021.1962637)
Supplement: Supplemental Material [file KCCY_A_1962637_SM1576.zip › Supplementary information/Rezig et al S2 Table.docx]

S2 Table. Vector DNA constructs used in this study. "GB" number refers to the laboratory reference collection.

| **GB No.** | **Abbreviated plasmid vector description** |
| --- | --- |
| 880 | pET-14b - His tagged, Vps4p full length, *Nde* I*/Bam* HI  Invitrogen order 13AB6ZFP |
| 881 | pGEX4T1 - GST tagged, Mid1p 1-453, *Bam* HI*/Xho* I - “N-term”  GenScript order U2640BJ110 |
| 882 | pGEX4T1 - GST tagged, Mid1p 452-579, *Bam* HI*/Xho* I - “Middle” |
| 883 | pGEX4T1 - GST tagged, Mid1p 578-799, *Bam* HI*/Xho* I |
| 884 | pGEX4T1 - GST tagged, Mid1p 798-920, *Bam* HI*/Xho* I - “C-term” |
| 889 | pET-14b - His tagged, Myo2p C-terminus amino acids 1394-1526, *Nde* I*/Bam* HI  GenScript order U9540CD270_2 |
| 907 | *mid1*^+^ wild-type + 1 kb upstream of ORF - 3,853 base pairs in total  *Kpn* I/*Sac* I fragment cloned into pJK148  GenScript order U2002DH100 |
| 909 | *mid1* mutant (1) S523 to A523 in pJK148 |
| 911 | *mid1* mutant (1) S523 to D523 in pJK148 |
| 913 | *mid1* mutant (1) S531 to A531 in pJK148 |
| 915 | *mid1* mutant (1) S531 to D531 in pJK148 |
| 917 | *mid1* mutant (2) S523 to A523 + S531 to A531 in pJK148 |
| 919 | *mid1* mutant (2) S523 to D523 + S531 to D531 in pJK148 |
| 923 | *mid1* mutant (1) S167 to A167 in pJK148  GenScript order U894VEB050 |
| 924 | *mid1* mutant (1) S167 to D167 in pJK148 |
| 925 | *mid1* mutant (1) S328 to A328 in pJK148 |
| 926 | *mid1* mutant (1) S328 to D328 in pJK148 |
| 927 | *mid1* mutant (1) S331 to A331 in pJK148 |
| 928 | *mid1* mutant (1) S331 to D331 in pJK148 |
| 929 | *mid1* mutant (1) S332 to A332 in pJK148 |
| 930 | *mid1* mutant (1) S332 to D332 in pJK148 |
| 931 | *mid1* mutant (4) S167 to A167 + S328 to A328 + S331 to A331+ S332 to A332 in pJK148 |
| 932 | *mid1* mutant (4) S167 to D167 + S328 to D328 + S331 to D331+ S332 to D332 in pJK148 |
| 933 | *mid1* mutant (6) S167 to A167 + S328 to A328 + S331 to A331+ S332 to A332 + S523 to A523 + S531 to A531 in pJK148 |
| 934 | *mid1* mutant (6) S167 to D167 + S328 to D328 + S331 to D331+ S332 to D332 + S523 to D523 + S531 to D531 in pJK148 |
| 935 | pGEX4T1 - GST tagged, Mid1p 1-453, *Bam* HI*/Xho* I - “N-term” S167 to A167  GenScript order U8198EF120 |
| 936 | pGEX4T1 - GST tagged, Mid1p 1-453, *Bam* HI*/Xho* I - “N-term” S328 to A328 |
| 937 | pGEX4T1 - GST tagged, Mid1p 1-453, *Bam* HI*/Xho* I - “N-term” S331 to A331 |
| 938 | pGEX4T1 - GST tagged, Mid1p 1-453, *Bam* HI*/Xho* I - “N-term” S332 to A332 |
| 939 | pGEX4T1 - GST tagged, Mid1p 1-453, *Bam* HI*/Xho* I - “N-term”  S167 to A167 + S328 to A328 + S331 to A331 + S332 to A332 |
| 940 | pGEX4T1 - GST tagged, Mid1p 452-579, *Bam* HI*/Xho* I - “Middle” S523 to A523 |
| 941 | pGEX4T1 - GST tagged, Mid1p 452-579, *Bam* HI*/Xho* I - “Middle” S531 to A531 |
| 942 | pGEX4T1 - GST tagged, Mid1p 452-579, *Bam* HI*/Xho* I - “Middle”  S523 to A523 + S531 to A531 |
