# Supplementary material for: Anillin/Mid1p interacts with the ESCRT-associated protein Vps4p and mitotic kinases to regulate cytokinesis in fission yeast
Source: Cell Cycle. 2021 Aug 12;20(18):1845–60. doi: 10.1080/15384101.2021.1962637 (PMC8525990; doi:10.1080/15384101.2021.1962637)
Supplement: Supplemental Material [file KCCY_A_1962637_SM1576.zip › Supplementary information/Rezig et al S3 Table.docx]

S3 Table. Summary of *S. pombe* global proteomic studies of identified Mid1p phospho-sites.

| **Study** | **Total number of phosphory-lation events** | **Mid1p phospho-acceptor sites** | | | | **Reference** |
| --- | --- | --- | --- | --- | --- | --- |
|  |  | Ref. No. | **Mid1p** **Residue** **position** | Ref. No. | **Mid1p** **Residue** **position** |  |
| **1** | 8000 | 7140 | **S15** | 7150 | **S335** | [30] |
|  |  | 7141 | **S18** | 7151 | **S110** |  |
|  |  | 7142 | **S167** | 7152 | **S112** |  |
|  |  | 7144 | **S24** | 7153 | **S344** |  |
|  |  | 7145 | **S27** | 7154 | **S347** |  |
|  |  | 7146 | **S328** | 9637 | **T336** |  |
|  |  | 7148 | **S331** | 9638 | **T111** |  |
|  |  | 7149 | **S332** | 10,040 | **Y333** |  |
| **2** | 3682 | 3150 | **S7** | 3156 | **S328** | [31] |
|  |  | 3151 | **S167** | 3157 | **S331** |  |
|  |  | 3153 | **S24** | 3158 | **S109** |  |
|  |  | 3154 | **S27** | 3159 | **S344** |  |
|  |  | 3155 | **28** | 3160 | **S347** |  |
|  |  |  |  | 20,982 | **S523** |  |
| **3** | 12,524 | 10,736 | **S218** | 10,749 | **S434** | [32] |
|  |  | 10,738 | **S24** | 10,750 | **T435** |  |
|  |  | 5831 | **S167** | 10,755 | **S440** |  |
|  |  | 10,742 | **S331** | 10,756 | **S444** |  |
|  |  | 10,743 | **S328** | 10,757 | **S445** |  |
|  |  | 10,744 | **S332** | 10,758 | **S532** |  |
|  |  | 10,745 | **T336** | 10,759 | **S527** |  |
|  |  | 10,746 | **S403** | 10,760 | **S531** |  |
|  |  | 10,747 | **S432** | 10,764 | **T34** |  |
|  |  | 10,748 | **S433** | 10,765 | **S42** |  |
|  |  |  |  | 10,767 | **S335** |  |
| **4** | 7298 | 5835 | **S167** | 5844 | **S403** | [33] |
|  |  | 5836 | **S218** | 5845 | **S434** |  |
|  |  | 5837 | **S24** | 5846 | **S444** |  |
|  |  | 5838 | **S27** | 5847 | **S531** |  |
|  |  | 5839 | **S28** | 5848 | **S541** |  |
|  |  | 5840 | **S328** | 5849 | **S7** |  |
|  |  | 5842 | **S331** | 5850 | **T34** |  |
|  |  | 5843 | **S395** | 5851 | **T405** |  |
