# Supplementary material for: Anillin/Mid1p interacts with the ESCRT-associated protein Vps4p and mitotic kinases to regulate cytokinesis in fission yeast
Source: Cell Cycle. 2021 Aug 12;20(18):1845–60. doi: 10.1080/15384101.2021.1962637 (PMC8525990; doi:10.1080/15384101.2021.1962637)
Supplement: Supplemental Material [file KCCY_A_1962637_SM1576.zip › Supplementary information/Rezig et al S4 Table.docx]

S4 Table. Summary of *S. pombe* cell morphology/growth synthetic phenotypes of single/double *mid1* phospho-acceptor site mutants combined with *plo1*-*ts35*, *ark1*-*T11* and *vps4*∆ mutants. In each case strains containing serine residues (S167, S328, S331, S332, S523 or S531) in Mid1 were converted to either a phospho-mimetic (S>D) or a phospho-resistant (S>A) point mutation(s) of the residues to create a panel of mutant *S. pombe* strains. Such *mid1* mutations were made both singly and in combination. Each was then combined individually with the mutants *plo1*-*ts35*, *ark1*-*T11* or *vps4*∆ with cell morphology and growth analysed of the double mutants when grown on solid medium to identify synthetic phenotypes. Double mutants displaying phenotypes mentioned in the text are circled in red. Note that the *mid1* S331A mutant was not created as cells were found to be sterile.

| *mid1* mutations  S = serine  A = adenine  D = aspartic acid  Single or in combination | | *mid1* single/double phospho-receptor mutant synthetic phenotypes  Morph= cell morphology, WT = wild-type, *mid1*∆ = *mid1* deletion, *ark1* = *ark1*-*T11*, Int. = intermediate between WT and *mid1*∆ | | | | | | | | GG  No. |
| --- | --- | --- | --- | --- | --- | --- | --- | --- | --- | --- |
|  |  | *mid1* single mutant | | *mid1* *plo1*-*ts35* double mutant | | *mid1* *ark1*-*T11* double mutant | | *mid1* *vps4*∆  double mutant | |  |
|  |  | Morph | Growth | Morph | Growth | Morph | Growth | Morph | Growth |  |
| *mid1* wild-type | | WT | WT | WT | Int. | WT | WT | WT | WT | 3181 |
| *mid1* S167 (1) | A | WT | WT | WT | WT | WT | WT | WT | WT | 3267 |
|  | D | WT | WT | WT | WT | WT | Int. | WT | WT | 3271 |
| *mid1* S328 (1) | A | WT | slow | WT | WT | WT | WT | WT | WT | 3275 |
|  | D | WT | WT | WT | WT | WT | Int. | WT | WT | 3280 |
| *mid1* S331 (1) | D | WT | WT | WT | WT | WT | Int. | WT | WT | 3283 |
| *mid1* S332 (1) | A | *mid1*∆ | *mid1*∆ | *mid1*∆ | *mid1*∆ | *mid1*∆ | *mid1*∆ | *mid1*∆ | *mid1*∆ | 3291 |
|  | D | WT | WT | Int. | WT | WT | WT | WT | WT | 3295 |
| *mid1* S523 (1) | A | WT | WT | WT | WT | WT | Int. | WT | WT | 3185 |
|  | D | WT | WT | WT | WT | WT | WT | WT | WT | 3189 |
| *mid1* S531 (1) | A | WT | WT | WT | Int. | *ark1* | *mid1*∆ | WT | WT | 3193 |
|  | D | WT | WT | WT | Int. | *ark1* | *mid1*∆ | WT | WT | 3197 |
| *mid1* S167-S332 (4) | A | *mid1*∆ | *mid1*∆ | *mid1*∆ | *mid1*∆ | *mid1*∆ | *mid1*∆ | *mid1*∆ | *mid1*∆ | 3299 |
|  | D | WT | WT | Int. | WT | WT | WT | WT | WT | 3305 |
| *mid1* S523-S531 (2) | A | *mid1*∆ | *mid1*∆ | *mid1*∆ | *mid1*∆ | *mid1*∆ | *mid1*∆ | *mid1*∆ | slow | 3201 |
|  | D | WT | WT | Int. | WT | WT | Int. | WT | WT | 3205 |
| *mid1* S167-S531 (6) | A | *mid1*∆ | *mid1*∆ | *mid1*∆ | *mid1*∆ | *mid1*∆ | *mid1*∆ | *mid1*∆ | *mid1*∆ | 3307 |
|  | D | WT | WT | WT | WT | WT | WT | WT | WT | 3311 |
