# Supplementary material for: Anillin/Mid1p interacts with the ESCRT-associated protein Vps4p and mitotic kinases to regulate cytokinesis in fission yeast
Source: Cell Cycle. 2021 Aug 12;20(18):1845–60. doi: 10.1080/15384101.2021.1962637 (PMC8525990; doi:10.1080/15384101.2021.1962637)
Supplement: Supplemental Material [file KCCY_A_1962637_SM1576.zip › Supplementary information/Rezig_et_al_S5_Table.docx]

S5 Table. Reduction of *in* *vitro* phosphorylation signal for Mid1-N term by Aurora A and Plk1 kinases in triplicate experiments, red coloured samples show significant decrease of *in vitro* phosphorylation.

| **Sample** | **Percentage of reduction compared to Mid1 N-term sample** | **P-value (*2-tailed*)** |
| --- | --- | --- |
| ***in* *vitro* phosphorylation by Aurora A** | | |
| Mid1 N-term vs. Mid1 N-term S167A | 91.84 % | <0.0001 |
| Mid1 N-term vs. Mid1 N-term S328A | 46.36 % | 0.009 |
| Mid1 N-term vs. Mid1 N-term S331A | 54.79 % | 0.004 |
| Mid1 N-term vs. Mid1 N-term S332A | 51.61 % | 0.006 |
| Mid1 N-term vs. Mid1 N-term Multiple | 97.89 % | <0.0001 |
| ***in* *vitro* phosphorylation by Plk1** | | |
| Mid1 N-term vs. Mid1 N-term S167A | 92.77% | <0.0001 |
| Mid1 N-term vs. Mid1 N-term S328A | 62.03% | 0.007 |
| Mid1 N-term vs. Mid1 N-term S331A | 91.86% | <0.0001 |
| Mid1 N-term vs. Mid1 N-term S332A | 62.40% | 0.041 |
| Mid1 N-term vs. Mid1 N-term Multiple | 91.25% | <0.0001 |
