# Supplementary figures and images for: Anillin/Mid1p interacts with the ESCRT-associated protein Vps4p and mitotic kinases to regulate cytokinesis in fission yeast
Source: Cell Cycle. 2021 Aug 12;20(18):1845–60. doi: 10.1080/15384101.2021.1962637 (PMC8525990; doi:10.1080/15384101.2021.1962637)

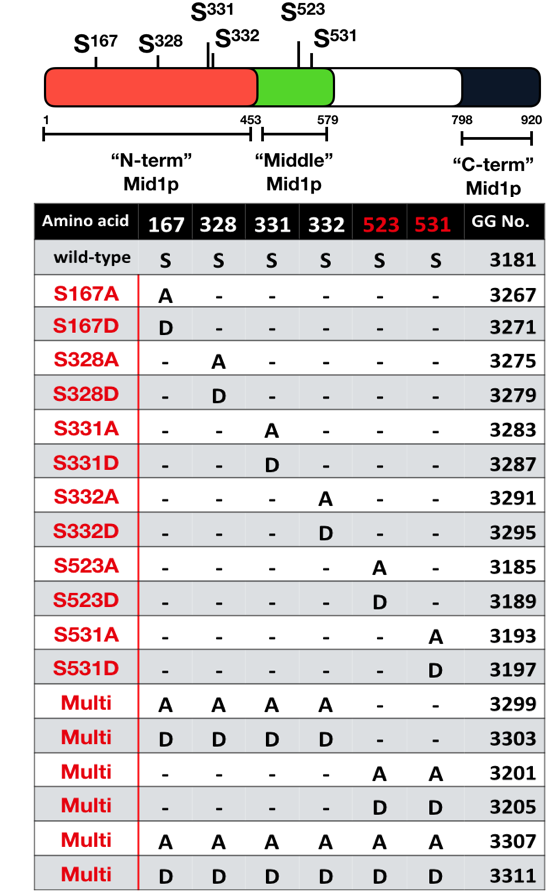

Supplement: Supplemental Material [file KCCY_A_1962637_SM1576.zip › Supplementary information/Rezig et al S1 Fig.tiff]

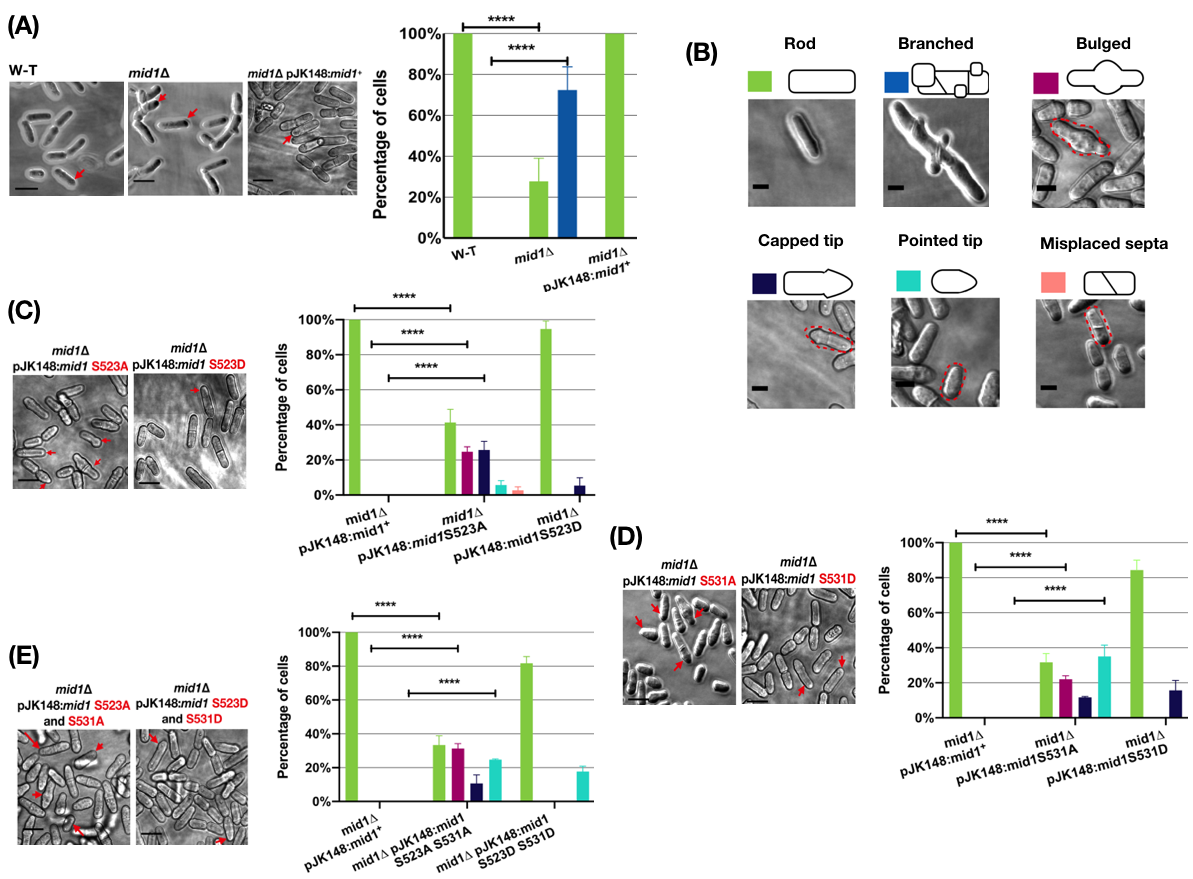

Supplement: Supplemental Material [file KCCY_A_1962637_SM1576.zip › Supplementary information/Rezig et al S2 Fig.tiff]

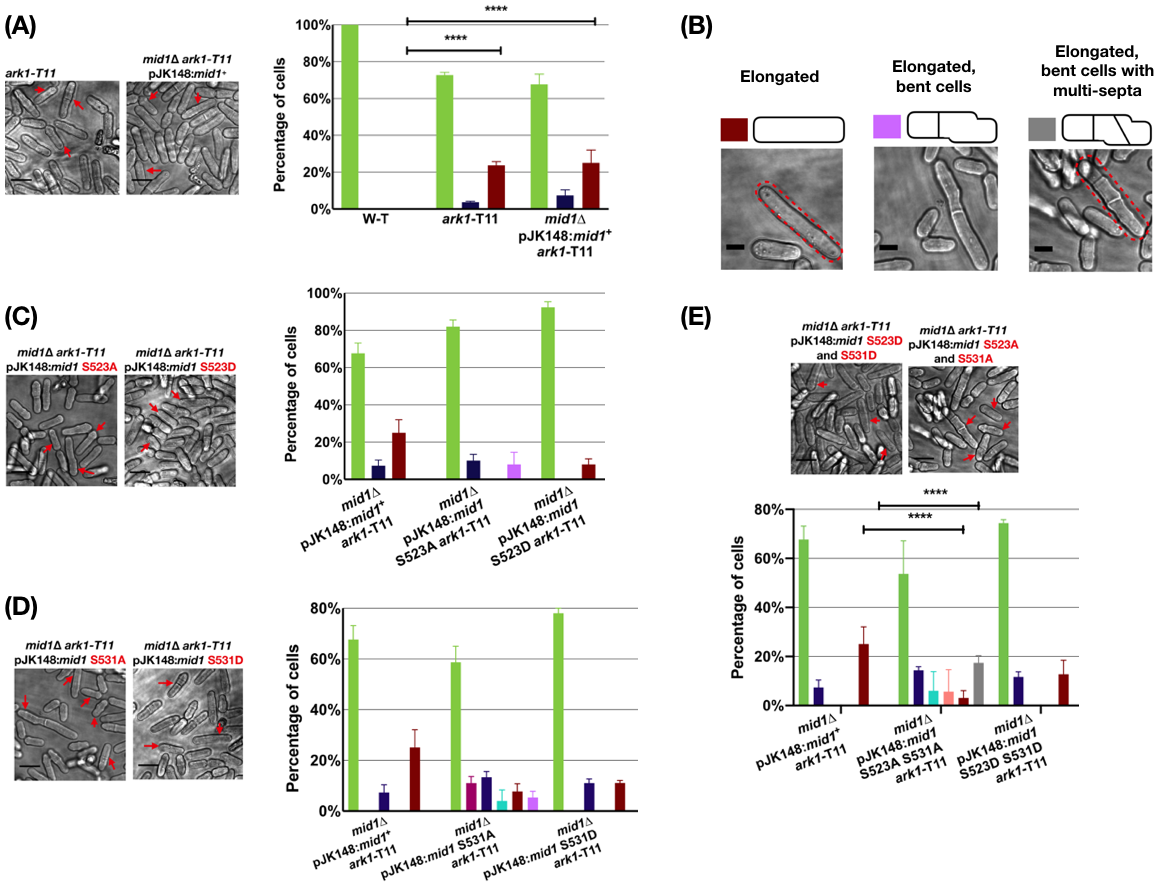

Supplement: Supplemental Material [file KCCY_A_1962637_SM1576.zip › Supplementary information/Rezig et al S3 Fig.tiff]

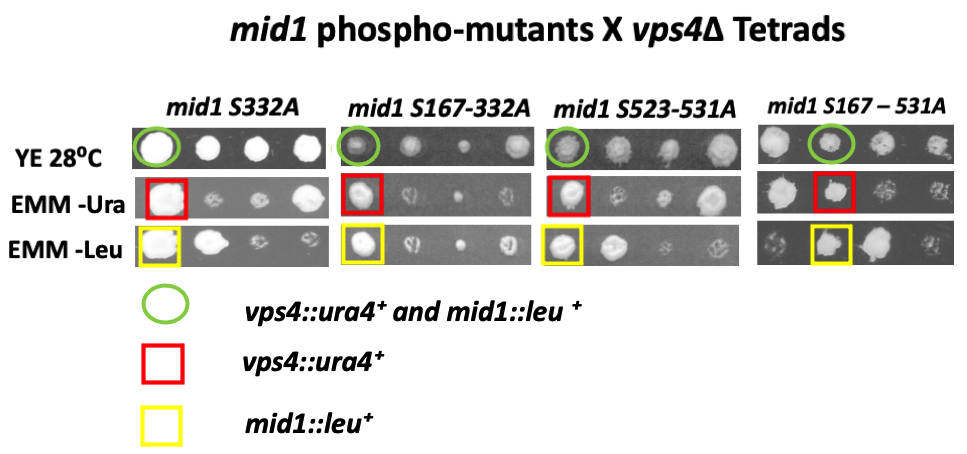

Supplement: Supplemental Material [file KCCY_A_1962637_SM1576.zip › Supplementary information/Rezig et al S4 Fig.tiff]

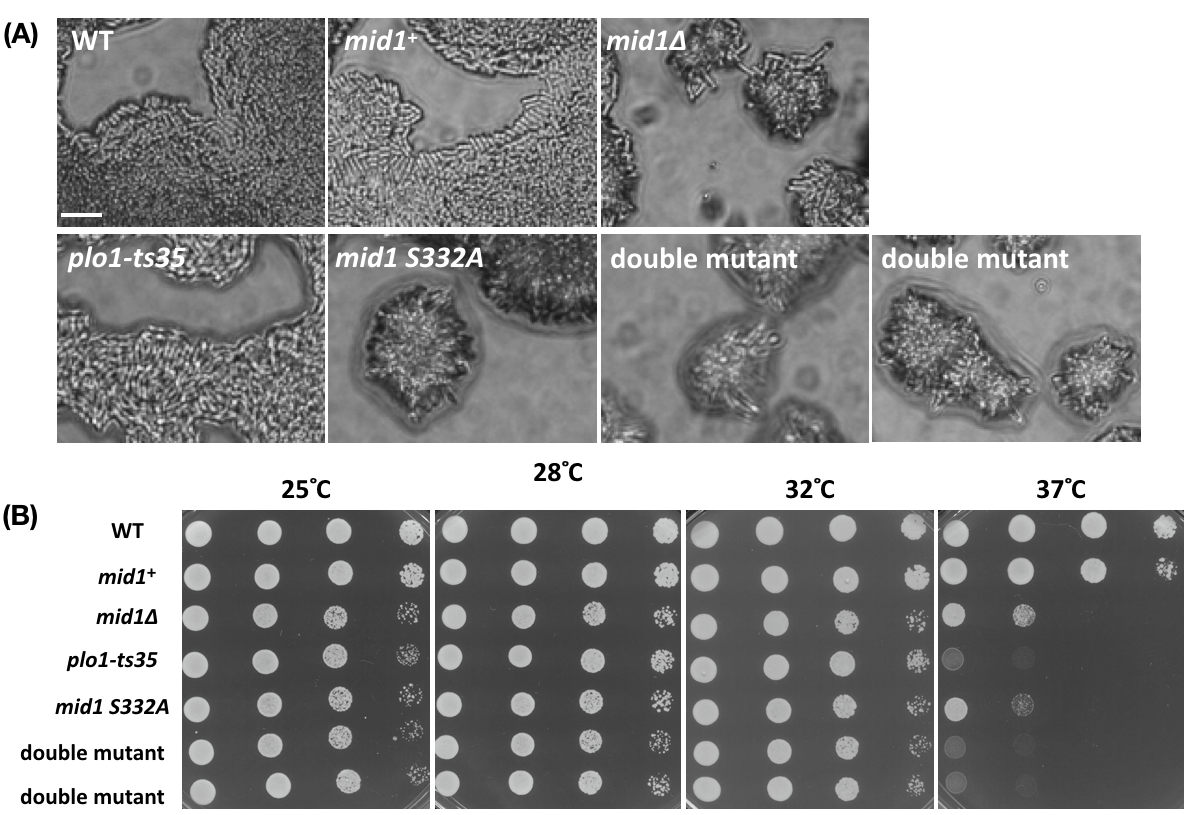

Supplement: Supplemental Material [file KCCY_A_1962637_SM1576.zip › Supplementary information/Rezig et al S5 Fig.tiff]
